# A new species of *Cretalamna sensu stricto* (Lamniformes, Otodontidae) from the Late Cretaceous (Santonian-Campanian) of Alabama, USA

**DOI:** 10.7717/peerj.4229

**Published:** 2018-01-08

**Authors:** Jun A. Ebersole, Dana J. Ehret

**Affiliations:** 1 McWane Science Center, Birmingham, AL, USA; 2 University of Alabama, Alabama Museum of Natural History, Tuscaloosa, AL, USA; 3 Current affiliation: New Jersey State Museum, Trenton, USA

**Keywords:** Chondrichthyes, Elasmobranchii, Mooreville Chalk, Tombigbee Sand Member, Lamniformes

## Abstract

Decades of collecting from exposures of the Upper Cretaceous Tombigbee Sand Member of the Eutaw Formation and Mooreville Chalk in Alabama, USA has produced large numbers of isolated *Cretalamna* (*sensu stricto*) teeth. Many of these teeth had formerly been assigned to the extinct Late Cretaceous shark *Cretalamna appendiculata* (Agassiz, 1843), a taxon that is now considered largely restricted to the Turonian of Europe. Recent studies have shed light on the diversity of Late Cretaceous *Cretalamna* (*s.s*.) taxa, and here we recognize a new species from Alabama, *Cretalamna bryanti*. The teeth of *C. bryanti* sp. nov. appear aligned with the members of the *Cretalamna borealis* species group, but can be distinguished from these other species by a combination of the following: anterior teeth with a more pronounced and triangular lingual root protuberance, broader triangular cusp, and a taller root relative to the height of the crown; anteriorly situated lateroposterior teeth have a distally inclined or hooked main cusp and more than one pair of lateral cusplets; and lateroposterior teeth have a strong distally hooked main cusp and a root that is largely symmetrical in basal view. At present, *C. bryanti* sp. nov. is stratigraphically confined to the Santonian/Campanian *Dicarinella asymetrica* Sigal, 1952 and *Globotruncanita elevata* Brotzen, 1934 Planktonic Foraminiferal Zones within the Tombigbee Sand Member of the Eutaw Formation and Mooreville Chalk, and teeth have been collected from only four counties in central and western Alabama. The recognition of *C. bryanti* sp. nov. in Alabama adds to our knowledge on the diversity and distribution of Late Cretaceous otodontids in the region.

## Introduction

*Cretalamna* is an extinct genus of lamniform shark that has been described globally from deposits ranging from the Albian (Early Cretaceous) to the Ypresian (Early Eocene) (Cappetta, 2012; Siversson et al., 2015). Glickman (1958) originally designated *Otodus appendiculatus* Agassiz, 1843 the type species for *Cretalamna*, however Siverson (1999) and Siversson et al. (2015) have since noted issues with Agassiz’s (1843) original syntypes. Not only did Agassiz (1843) not designate a holotype, but the specimens he figured appeared to represent a heterogeneous mix of several different genera (Siversson et al., 2015).

The compilation of taxa that comprised Agassiz’s (1843) original syntypes subsequently resulted in a broad interpretation regarding the morphology of *C. appendiculata*, consequently leading it to become a ‘waste basket’ taxon. To help remedy this situation, Siverson (1999) designated one of Agassiz’s (1843) former syntypes (pl. 32, fig. 10) the lectotype for *C. appendiculata* (*sensu stricto).* Later, Siversson et al. (2015) lent clarity to the diversity of Late Cretaceous *Cretalamna*-like species when they provided an amended diagnosis for *C. appendiculata* (*s.s*), and identified numerous *C. appendiculata*-like taxa spanning from the Cenomanian through the Campanian. Based on similarities in tooth morphology, Siversson et al. (2015) established three species groups for all but two of these *Cretalamna* (*s.s.*) species, the *Cretalamna appendiculata* group, the *Cretalamna borealis* group, and the *Cretalamna hattini* group.

In Alabama, decades of collecting from exposures of the Upper Cretaceous Tombigbee Sand Member of the Eutaw Formation and Mooreville Chalk has produced large numbers of documented *Cretalamna* teeth (Applegate, 1970; Thurmond & Jones, 1981; Ikejiri et al., 2013; Ciampaglio et al., 2013). Historically, most of these isolated teeth were assigned to *C. appendiculata* (see Applegate, 1970; Thurmond & Jones, 1981; Russell, 1988; Ciampaglio et al., 2013; Ikejiri et al., 2013), a taxon that is now considered largely confined to the Turonian of Europe (Siversson et al., 2015). The present study is the result of a reexamination of these *Cretalamna* teeth and their comparison to the various *C. appendiculata*-like species described by Siversson et al. (2015). This comparison has culminated in the recognition of a new species of *Cretalamna* (*s.s.*) from the Late Cretaceous of Alabama.

**Table 1:** *Cretalamna bryanti* sp. nov. specimens identified as part of this study. (ALMNH) Alabama Museum of Natural History, Tuscaloosa, USA. (MSC) McWane Science Center, Birmingham, Alabama, USA. (CH) crown height. (CT) crown thickness. (CW) main cusp width. (TH) total height. (TW) total width. (N/A) measurement could not be taken due to specimen breakage. All measurements are in millimeters (mm).

Catalog Number	Stratigraphic unit	Locality	Tooth group	CH (mm)	TH (mm)	CW (mm)	TW (mm)	CT (mm)	Date collected	Comments
ALMNH 1068	Lower Mooreville Chalk	Site ADa-3	Upper right lateroposterior	14.1	16.9	8.4	N/A	4.3	6/28/1988	Mesial root lobe and cusplets missing, apex of crown worn
ALMNH 1164	Lower Mooreville Chalk	Site ADa-3	Upper left anteriorly situated lateroposterior; large morphology	12.8	N/A	11.8	23.3	4.9	6/20/1988	Entire apex broken
ALMNH 1407	Lower Mooreville Chalk	Site ADa-3	Upper right anterior	16.6	21.7	6.9	13.6	3.4	7/8/1988	distal cusplet broken off
ALMNH 1682	Lower Mooreville Chalk	Site ADa-3	Lower right anterior	19.6	24.8	8.5	14.2	4.6	7/8/1988	Distal root lobe broken
ALMNH 1719	Lower Mooreville Chalk	Site ADa-3	Lower left anterior	18.1	23.8	8.9	16	4.2	6/23/1988	****
ALMNH 3322	Lower Mooreville Chalk	Site ADa-3	Upper right anterior	18.7	23.8	7.7	14.4	3.6	1993	**Paratype**
ALMNH 3330	Lower Mooreville Chalk	Site ADa-19	Upper left anteriorly situated lateroposterior	15.3	21.1	10.5	20.1	4.2	6/30/1990	
ALMNH 3331	Lower Mooreville Chalk	Site ADa-20	Upper right lateroposterior	11.4	16.9	9.1	18	3.5	7/6/1993	
ALMNH 3566	Lower Mooreville Chalk	Site ADa-20	Upper right anterior	21.5	26.6	9.6	16.4	5.1	6/20/1993	
ALMNH 3935	Lower Mooreville Chalk	Site AGr-5	Upper right anterior	25.2	33.8	11.3	20.5	6	6/22/1994	Broken root lobe
ALMNH 4190	Tombigbee Sand Member	Site AGr-43	Upper left lateroposterior	8.9	12.7	7.4	14.6	2.8	7/8/1994	
ALMNH 4517	Lower Mooreville Chalk	Site ADa-19	Upper left lateroposterior	11.5	15	7.8	15.4	3.5	7/10/1996	
ALMNH 5195.1	Lower unnamed member of the Mooreville Chalk	Site AGr-5	Upper left anteriorly situated lateroposterior; large morphology	19.6	24.5	13.4	18.4	5.6	6/8/2005	Mesial and distal roots damaged, distal cusplet broken
ALMNH 5195.2	Lower unnamed member of the Mooreville Chalk	Site AGr-5	Lower left anteriorly situated lateroposterior; large morphology	N/A	N/A	13.8	N/A	5.4	6/8/2005	Most of root and distal cusplets missing
ALMNH 5360	Lower unnamed member of the Mooreville Chalk	Site AGr-4	Upper right lateroposterior	10	14.7	7.6	15.7	3.3	6/15/2005	Apex of crown missing
ALMNH 6306	Lower Mooreville Chalk	Site ADa-3	Lower left anteriorly situated lateroposterior	16.2	20.8	9.1	18.5	4.8	2010	**Paratype**
ALMNH 6728	Lower Mooreville Chalk	AGr-Exp. 33	Upper left anteriorly situated lateroposterior; large morphology	17.3	22.5	11.5	22.8	4.8	6/18/2003	Mesial and distal root lobes abraded
ALMNH 6760	Lower Mooreville Chalk	AGr-Exp. 33	Upper right lateroposterior	9.7	12.7	7.1	15.1	3.1	6/27/2013	
ALMNH 8668	Lower Mooreville Chalk	Site ADa-3	Upper left anteriorly situated lateroposterior; large morphology	18.2	24.8	14.8	26.2	5	6/25/2014	
ALMNH 9216	Lower Mooreville Chalk	Site ADa-3	Lower right anteriorly situated lateroposterior; large morphology	15.8	22.8	11.8	23.4	4.7	9/13/2015	Apex of crown missing
ALMNH 9348	Mooreville Chalk	AGr-Exp. 35	Upper right anterior	17.6	22.6	9.3	18.5	4.1	6/16/2015	
ALMNH 9724	Mooreville Chalk	Site ADa-3	Lower right anterior	20.3	26.5	8.2	15.9	4.6	11/13/2016	****
ALMNH 9878	Mooreville Chalk	Site ADa-3	Lower right anterior	13.6	16.8	7.4	13.6	2.8	2/4/2017	
ALMNH 15245	Lower Mooreville Chalk	Site ADa-3	Upper right lateroposterior	12.6	16.3	7.5	15	3	1993/1994	distal cusplet chipped
ALMNH 15250	Tombigbee Sand Member	Site AGr-43	Upper right anterior	15.9	20	9.7	16.5	3.8	10/4/2017	
MSC 1139.9	Tombigbee Sand Member	Site AHl-1	Upper left anteriorly situated lateroposterior	13.6	18.3	10.2	18.9	3.1	7/17/1980	
MSC 2984.1	Lower Mooreville Chalk	Site ADa-3	Upper right lateroposterior	12.4	18.8	9.3	17.8	3.9	7/1/1982	**Holotype**
MSC 5698	Lower Mooreville Chalk	Site AGr-18	Upper left anteriorly situated lateroposterior	15	21	10.5	20.6	3.9	3/14/1983	
MSC 5768	Lower Mooreville Chalk	Site ADa-3	Upper left lateroposterior	12.2	18	8.6	16.51	3.2	7/5/1982	Distal root lobe broken
MSC 26121	Lower unnamed member of the Mooreville Chalk	Site AGr-4	Upper right 1st? anteriorly situated lateroposterior	15.8	21.9	10.5	20.5	4.3	6/10/2011	
MSC 34051	Lower Mooreville Chalk	Site ADa-20	Upper left anteriorly situated lateroposterior	15.2	21.9	10.4	23.1	4.1	8/16/2008	
MSC 37499	Lower unnamed member of the Mooreville Chalk	Site AMg-1	Upper left lateroposterior; large morphology	12.8	18.6	10.9	N/A	3.8	11/21/1993	Distal base of crown and distal root lobe missing
MSC 37711	Lower Mooreville Chalk	Site ADa-3	Upper left anteriorly situated lateroposterior; large morphology	20.2	26.8	14.2	26.3	5.9	5/31/2008	

Herein we describe this new morphology and diagnose it against the numerous species of Late Cretaceous and Paleogene *Cretalamna*-like taxa from Alabama and elsewhere. In addition, we provide discussions on the various degrees of heterodonty observed within its dentition, document its stratigraphic and geographic distributions in the state, and discuss its taxonomic significance.

### Material and methods

The teeth described in this study (*n* = 33) were identified by the current authors within the collections of the ALMNH and MSC. The specimens were collected from nine different localities in Alabama (see Table 1), all of which are referenced in this study by standard Alabama Paleontological Locality Numbers. Lithologic and stratigraphic information for each locality, or reference(s) containing this data, is provided in Appendix 1. However due to legalities regarding site access and protection, precise locality information for every collection site is not provided herein. Rather, this data is on file at both the ALMNH and MSC and is fully available to qualified researchers upon request. The precise localities for three specimens in our sample are unknown. Records associated with these teeth indicate they were collected from Mooreville Chalk gully exposures within the vicinity of the town of Clinton in Greene County, Alabama, USA. However, due to the number of gully sites within the area, it cannot be known for certain which exposure the teeth were derived. As a result, these three specimens are listed with the non-standard locality designations of AGr-Exp. 33 or AGr-Exp. 35 (see Table 1 and Appendix 1).

**Figure 1:**
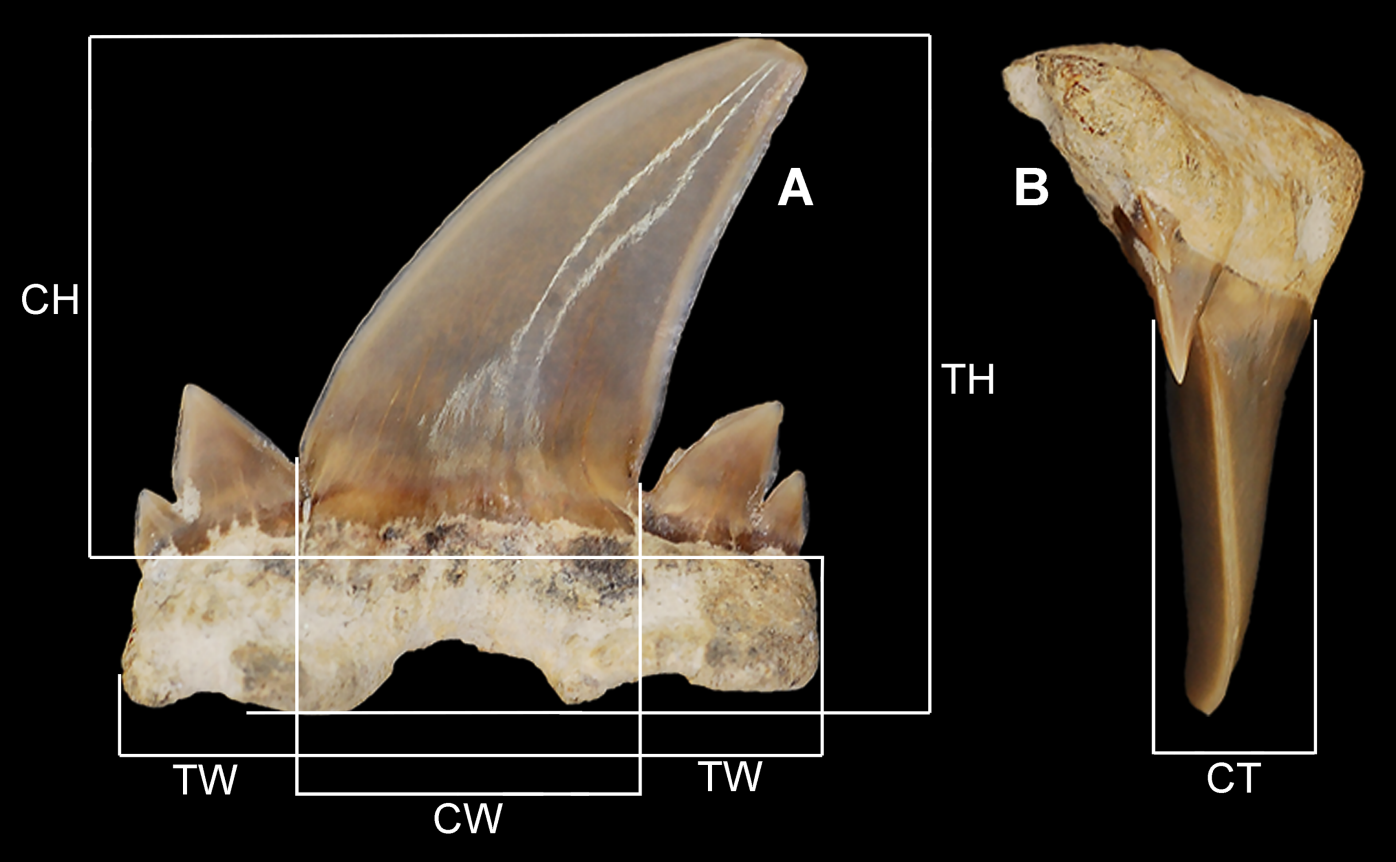
Diagram of tooth measurements taken as part of this study. MSC 2984.1, holotype in (A) labial view and (B) mesial view. (CH) crown height. (CT) crown thickness. (CW) main cusp width. (TH) total height. (TW) total width.

The examined specimens were collected from two types of localities, erosional gully exposures of Mooreville Chalk, and stream gravel bars containing vertebrate fossils derived from the Tombigbee Sand Member of the Eutaw Formation. The lone outlier is site AMg-1, a stream locality with exposures of both the Mooreville Chalk and Tombigbee Sand Member. Only one specimen (MSC 37499) was collected from this locality and it was derived from the Mooreville Chalk component exposed at this site. Most of the specimens (*n* = 26) were historically collected between the years 1980 and 2011. Because most of the examined specimens were not collected by the present authors, the direct methods of collection are not known for certain. However, after collecting at many of the same localities, the present authors can state with confidence that the specimens derived from localities consisting of erosional chalk gullies were obtained via surface collecting and those from stream localities were a result of either surface collection and/or screen washing matrix excavated from gravel bars.

Table 1 lists all the teeth examined as part of this study and includes the specimen repository, catalog number, locality number, stratigraphic unit, date collected, suggested tooth group, and tooth measurements. Standard measurements were taken of each tooth (Fig. 1), and notes on specimen breakage are reported when necessary. Because this new species is known only from isolated teeth, exact tooth positions within the jaw cannot be known for certain. Rather, the fossil teeth were placed within generalized tooth groups based on direct comparisons with extant jaw sets from various members of the Lamniformes (see Shimada, 2002). In particular, we examined jaw sets belonging to *Isurus oxyrincus* Rafinesque, 1810, *Isurus paucus* Guitart, 1966, and *Lamna nasus* (Bonnaterre, 1788) as these taxa have teeth with similar crown morphologies to those identified in this study. Teeth were placed into generalized anterior, anteriorly situated lateroposterior, and lateroposterior tooth groups based on a combination of distal crown inclination, number of lateral cusplets, and labial inclination of the roots (see the specific tooth group descriptions below for further discussions on these groupings). Commissural teeth are absent within our sample (likely due to a collecting bias), so teeth described herein from the lateroposterior files refer to those positioned within the inferred third lateroposterior hollow. Direct comparisons with extant jaw sets also assisted with the assigning of teeth to the upper or lower dentitions. On the extant jaw sets examined, teeth with a flat to lingually inclined main cusp were generally observed to occupy positions in the lower jaw (except for the first anterior tooth which has a weak lingual bend on the observed *I. oxyrincus I. paucus,* and *L. nasus* jaw sets). Teeth with a flat to labially inclined main cusp were generally observed to occupy the upper files.

The anatomical tooth terminology used in this study follows that of Shimada (2002), and tooth group terminology follows that of Siverson (1999). Higher taxonomic rankings used herein follow that of Nelson, Grande & Wilson (2016). Here we choose to follow Siversson et al. (2015) in the utilization of the spelling *Cretalamna* as opposed to *Cretolamna* (see Cappetta (2012) and Siversson et al. (2015) for competing arguments on the generic spelling). All specimens were photographed with a Nikon D80 Digital SLR camera with Tamron macro lens and rendered in Photoshop CC 2017 as part of the production of the presented figures. All specimens, including the holotype and paratype specimens, are housed in the scientific collections at either the ALMNH or MSC (see Table 1).

The electronic version of this article in Portable Document Format (PDF) will represent a published work according to the International Commission on Zoological Nomenclature (ICZN), and hence the new name contained in the electronic version are effectively published under that Code from the electronic edition alone. This published work and the nomenclatural acts it contains have been registered in ZooBank, the online registration system for the ICZN. The ZooBank LSIDs (Life Science Identifiers) can be resolved and the associated information viewed through any standard web browser by appending the LSID to the prefix http://zoobank.org/. The LSID for this publication is: urn:lsid:zoobank.org:pub:F6C65E1D-19E6-4BA9-BEDD-DAFDB0BC27C9. The online version of this work is archived and available from the following digital repositories: PeerJ, PubMed Central and CLOCKSS.”

## Geological Setting

**Figure 2:**
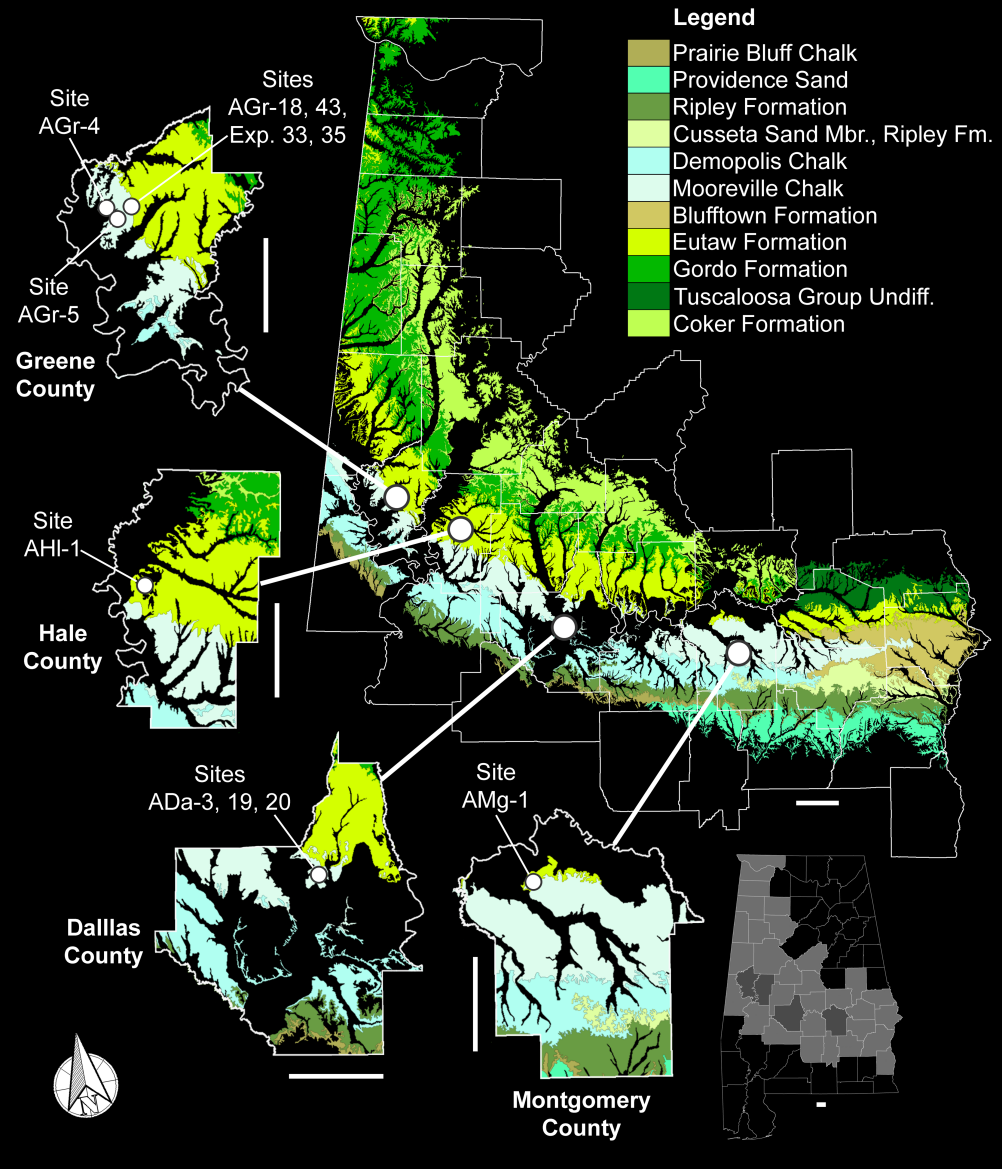
Upper Cretaceous surface exposures in Alabama and *Cretalamna bryanti* sp. nov. specimen collecting localities. Scale bars equal 20 km.

**Figure 3:**
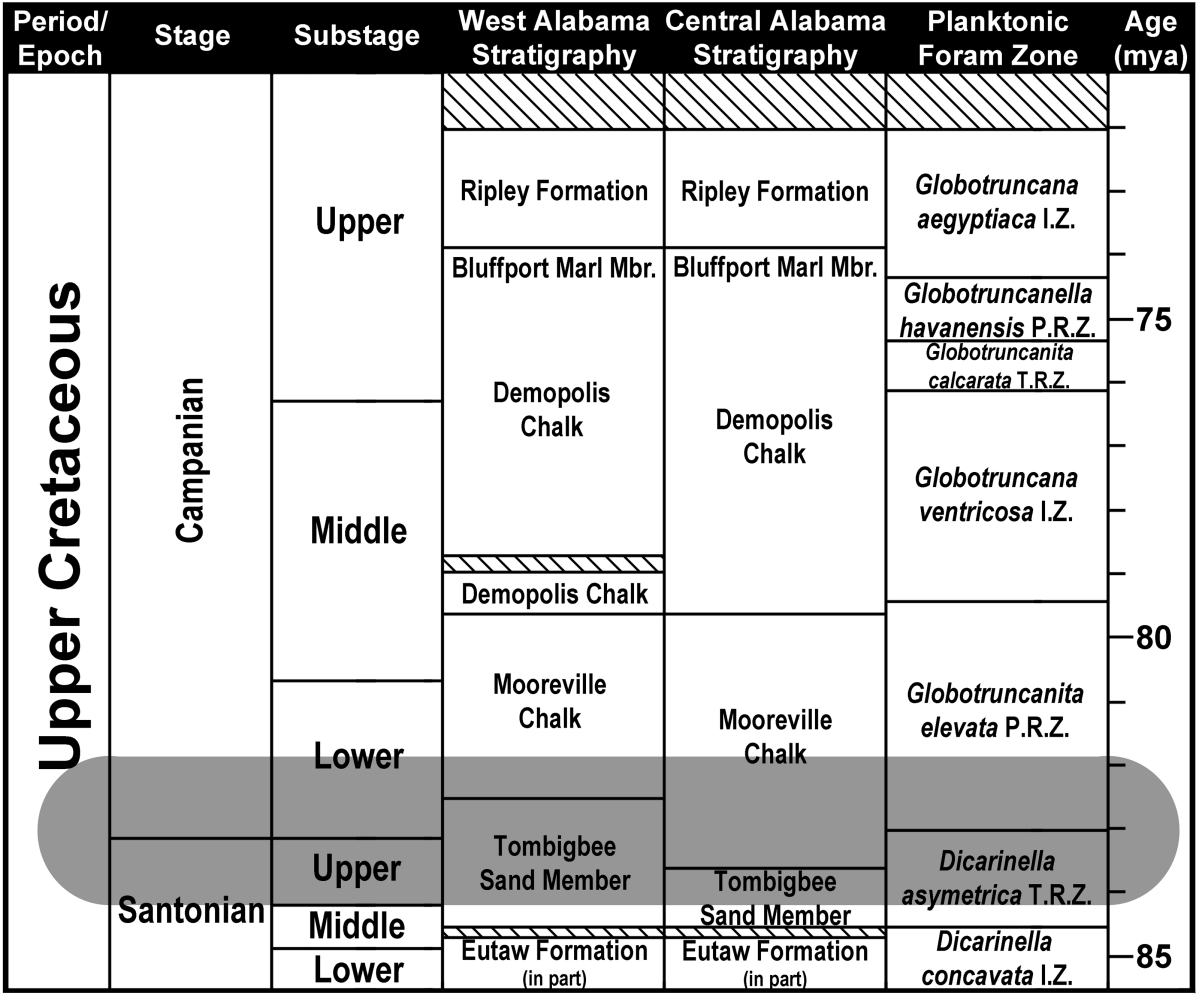
Santonian and Campanian surface stratigraphy in Alabama. Modified from Mancini, Puckett & Tew (1996). Striped areas represent unconformities. Shaded area represents the stratigraphic range of *Cretalamna bryanti* sp. nov. in Alabama. Planktonic Foraminifera Zones after Caron (1985).

The teeth described in this study were collected over the course of 30 years from nine distinct localities located within Dallas, Greene, Hale, and Montgomery counties in Alabama, USA (Fig. 2). These four counties are located within the center and western parts of a region in Alabama known locally as the Black Belt (Ebersole & Dean, 2013). In Alabama, the Black Belt is a 440-km wide stretch of land that extends east to west across the center of the state and into the northwest corner (Adams et al., 1926), forming the southeastern edge of the Late Cretaceous Mississippi Embayment. Stratigraphically, the surface exposures within this region form a nearly complete sequence of Upper Cretaceous strata that ranges from the Cenomanian to nearly the end of the Maastrichtian (Mancini, Puckett & Tew, 1996; Puckett, 2005). The examined specimens were derived from two stratigraphic units within this region, the Tombigbee Sand Member of the Eutaw Formation and the Mooreville Chalk (Fig. 3).

The Tombigbee Sand is the uppermost member of the Eutaw Formation. In central and western Alabama, the Eutaw Formation uncomformably overlies the Turonian/Coniacian Gordo Formation, and underlies the Mooreville Chalk. The Mooreville Chalk is comprised of two members, a lower unnamed member and the upper Arcola Limestone (Raymond et al., 1988; Mancini, Puckett & Tew, 1996; Puckett, 2005). The Mooreville Chalk lies conformably above the Tombigbee Sand Member, and below the upper Campanian Demopolis Chalk (which in conjunction with the Mooreville Chalk makes up the lower half of the Selma Group). In central and western Alabama, the contact between the Tombigbee Sand Member and the Mooreville Chalk is time transgressive and straddles the Santonian/Campanian boundary. In central Alabama, this contact resides within the late Santonian, whereas in west Alabama the contact between these two units falls within early Campanian (Mancini, Puckett & Tew, 1996; Puckett, 2005; Prieto-Márquez, Erickson & Ebersole, 2016b).

The lithology of the Tombigbee Sand Member is varied (Mancini & Soens, 1994), but the unit is largely composed of highly glauconitic sand that may include clay, sandy chalk, or calcareous sandstone, and has abundant burrows and invertebrate and vertebrate remains (Raymond et al., 1988). The base of this unit is indicative of a high energy, tidally influenced nearshore environment, but the upper portion of the Tombigbee Sand Member represents a low energy marine shelf environment (Mancini & Soens, 1994). A large majority of the Mooreville Chalk is made up of a light gray fossiliferous chalk and chalky marl, however the lower few meters of the unit, an unnamed member, is composed of glauconitic and clayey marl (Raymond et al., 1988). The combination of these lithologies suggests the depositional environment of the Mooreville Chalk to be a calm, middle-shelf environment with dysoxic bottom conditions (Wylie & King, 1986; Mancini & Soens, 1994).

**Figure 4:**
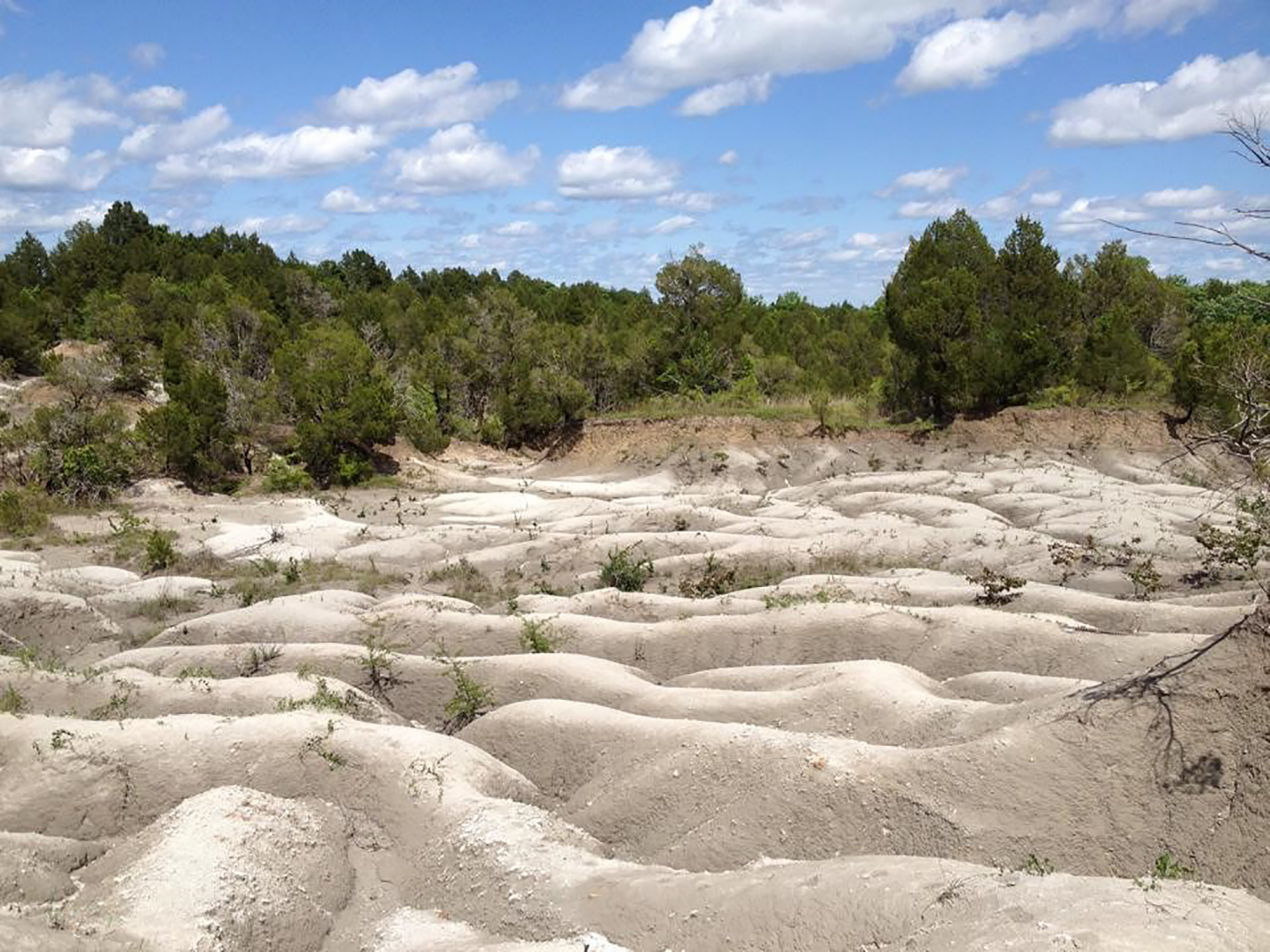
Mooreville Chalk gully exposures at the type section, site ADa-3 (Harrell Station), Dallas County, Alabama, USA. Photograph courtesy of Dana J. Ehret.

Although the examined specimens were collected from nine different localities, nearly half (15 of 33) were derived from the type locality, site ADa-3 in Dallas County, Alabama, USA (Fig. 2). Site ADa-3 is a 140-acre plot of land that is owned by the ALMNH and consists of extensive gully exposures of Mooreville Chalk, the only Late Cretaceous unit exposed at the site (Fig. 4). Studies by Puckett (2005) and Prieto-Márquez, Erickson & Ebersole (2016b) on the stratigraphy, ostracodes, planktonic foraminifera, and nannoflora at the site have shown that the Mooreville Chalk exposures at site ADa-3 fall within the *Globotruncanita elevata* Brotzen, 1934 Primary Foraminifera Range Zone (see Fig. 3), the upper half of the *Acuminobrachycythere acuminata* Hazel & Paulson, 1964 Ostracode Zone, and Nannofossil Zone CC-18a (Appendix 1). This places the exposures at this site within the Lower Mooreville Chalk with an age in the early, but not earliest, Campanian. Site ADa-3 is also the type locality for the hadrosauroid *Lophorhothon atopus* Langston, 1960. Detailed information on the other eight collecting localities can be found in Appendix 1.

## Systematic Paleontology

**Table:** 

Class Chondrichthyes Huxley, 1880
Subclass Euselachii Hay, 1902
Infraclass Elasmobranchii Bonaparte, 1838
Division Selachii Cope, 1871
Superorder Galeomorphii Compagno, 1973
Order Lamniformes Berg, 1958
Family Otodontidae Glikman, 1964
Genus *Cretalamna* Glikman, 1958

### Type species

*Otodus appendiculatus* Agassiz, 1843, Upper Cretaceous (within the Cenomanian to early Coniacian interval), Lewes, England.

**Table:** 

**Figure 5:**
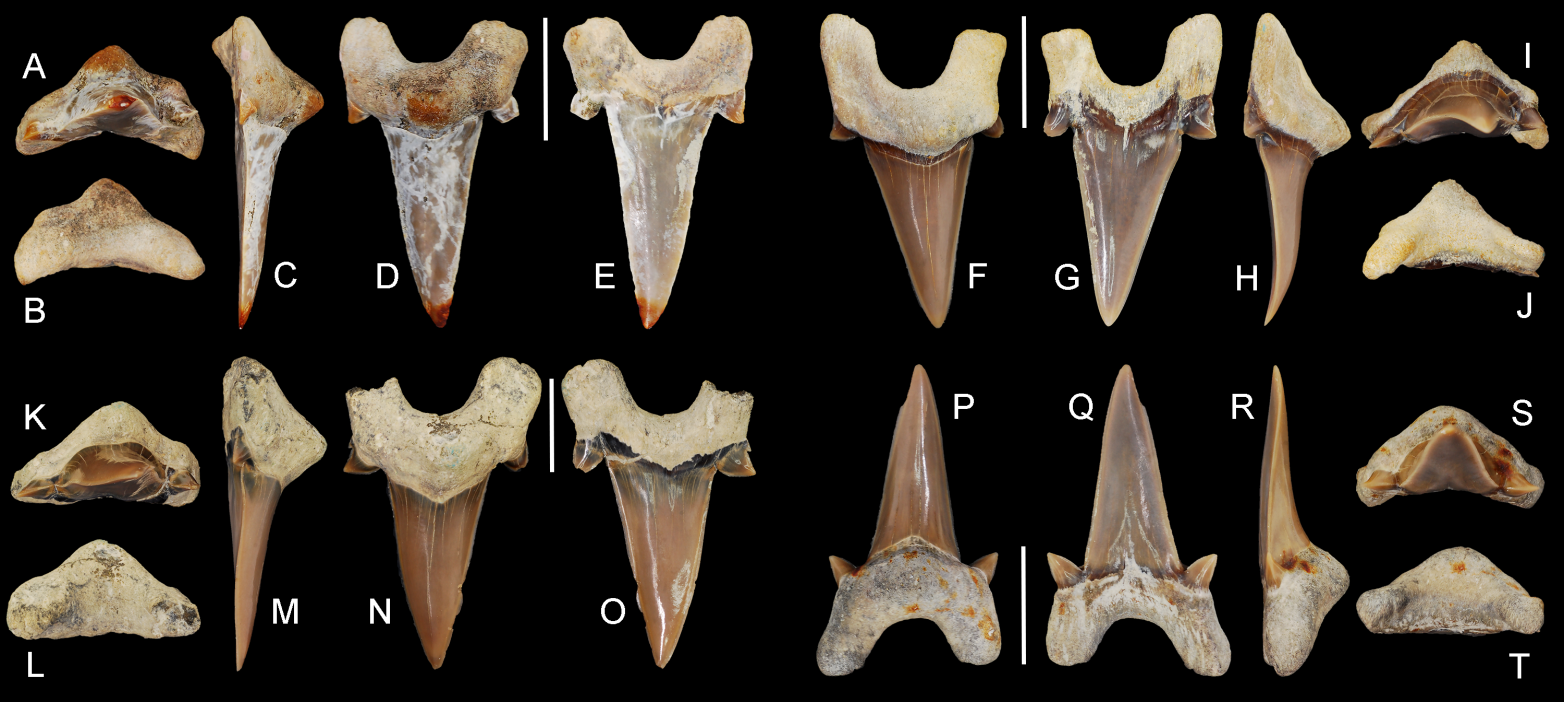
*Cretalamna bryanti* sp. nov. anterior teeth. (A–E) ALMNH 3322, paratype, upper right anterior tooth in (A) oral, (B) basal, (C) mesial, (D) lingual, and (E) labial views. (F–J) ALMNH 3566, upper right anterior tooth in (F) lingual, (G) labial, (H) mesial, (I) oral, and (J) basal views. (K–O) ALMNH 3935, upper left anterior tooth, large morphology, in (K) oral, (L) basal, (M) mesial, (N) lingual, and (O) labial views. (P–T) ALMNH 9724, lower right anterior tooth in (P) lingual, (Q) labial, (R) mesial, (S) oral, and (T) basal views. Scale bars equal 1.0 cm.

**Figure 6:**
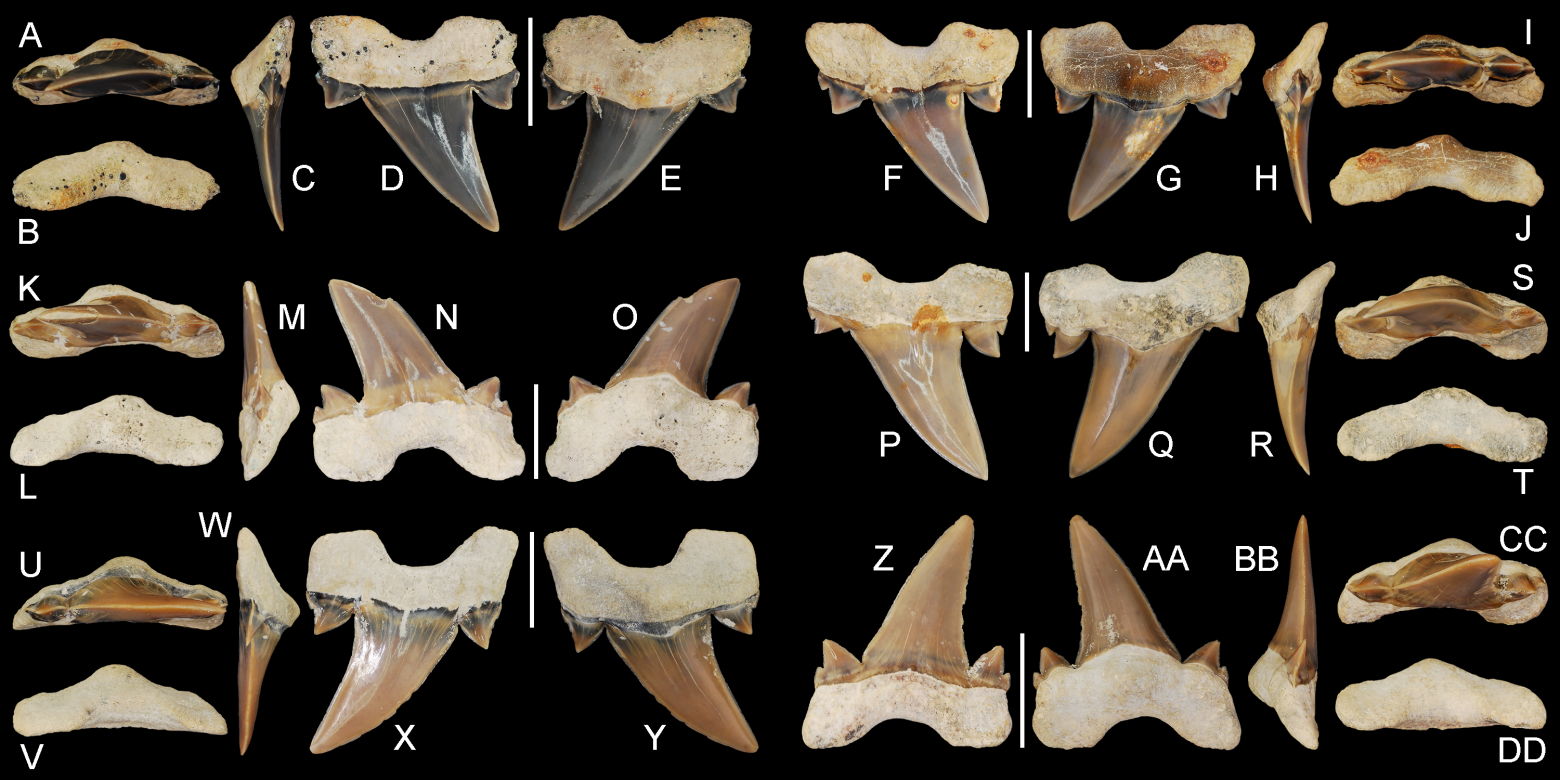
*Cretalamna bryanti* sp. nov. anteriorly situated lateroposterior teeth. (A–E) ALMNH 3330, upper left anteriorly situated lateroposterior tooth in (A) oral, (B) basal, (C) mesial, (D) labial, and (E) lingual views. (F–J) MSC 34051, upper left anteriorly situated lateroposterior tooth in (F) labial, (G) lingual, (H) mesial, (I) oral, and (J) basal views. (K–O) ALMNH 9216, lower right anteriorly situated lateroposterior tooth, large morphology in (K) oral, (L) basal, (M) mesial, (N) labial, and (O) lingual views. (P–T) MSC 37711, upper left anteriorly situated lateroposterior tooth, large morphology, in (P) labial, (Q) lingual, (R) mesial, (S) oral, and (T) basal views. (U-Y) MSC 26121, upper right anteriorly situated lateroposterior tooth in (U) oral, (V) basal, (W) mesial, (X) labial, and (Y) lingual views. (Z–AA) ALMNH 6306, paratype, lower left anteriorly situated lateroposterior tooth, in (Z) labial, (AA) lingual, (BB) mesial, (CC) oral, and (DD) basal views. Scale bars equal 1.0 cm.

**Figure 7:**
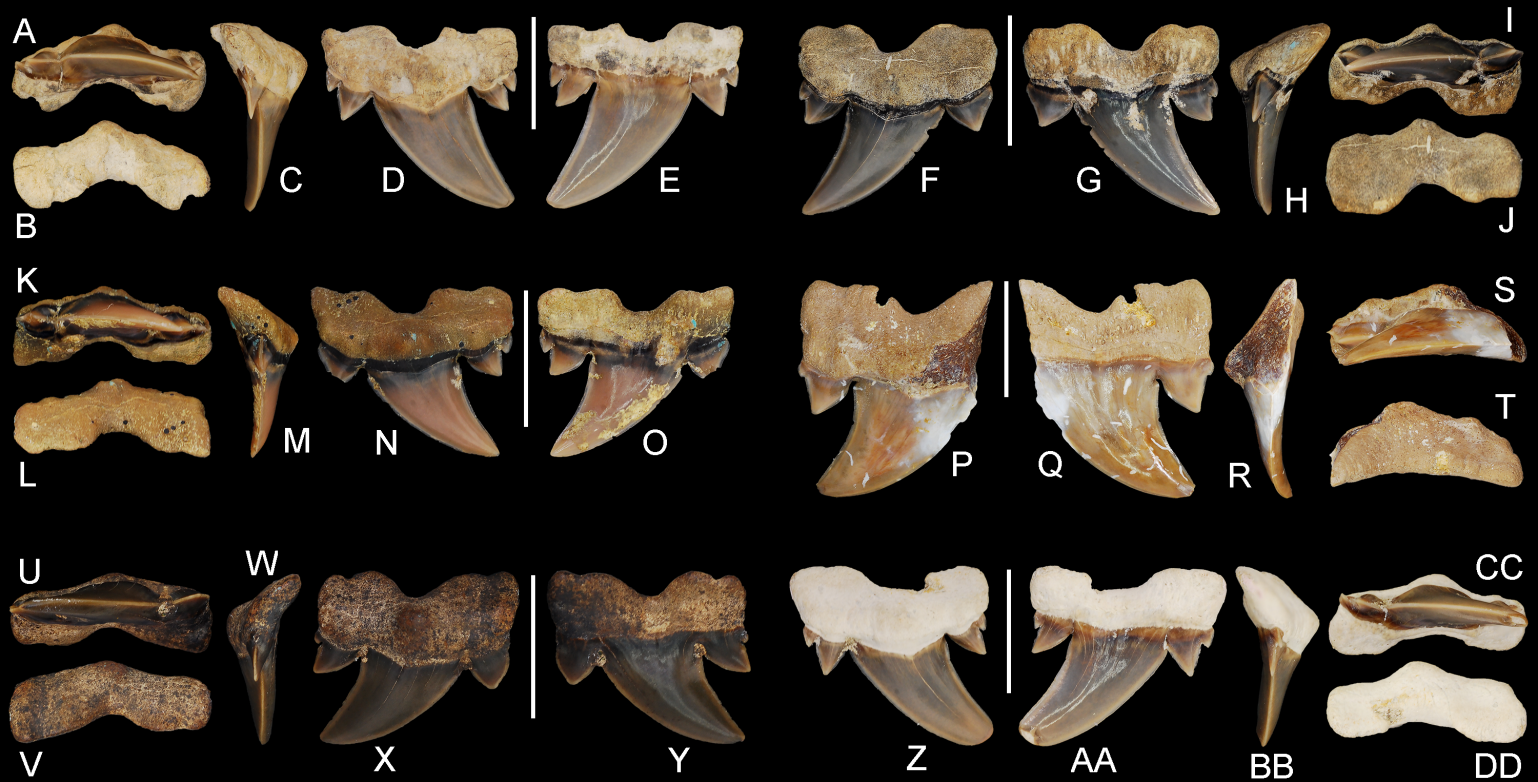
*Cretalamna bryanti* sp. nov. posteriorly situated lateroposterior teeth. (A–F) MSC 2984.1, holotype, upper right posteriorly situated lateroposterior tooth in (A) oral, (B) basal, (C) mesial, (D) lingual, and (E) labial views. (F–J) ALMNH 4517, upper left posteriorly situated lateroposterior tooth in (F) lingual, (G) labial, (H) mesial, (I) oral, and (J) basal views. (K–O) ALMNH 6760, upper right posteriorly situated lateroposterior tooth in (K) oral, (L) basal, (M) mesial, (N) lingual, and (O) labial views. (P–T) MSC 37499, upper left posteriorly situated lateroposterior tooth, large morphology, in (P) lingual, (Q) labial, (R) mesial, (S) oral, and (T) basal views. (U–Y) ALMNH 4190, upper left posteriorly situated lateroposterior tooth in (U) oral, (V) basal, (W) mesial, (X) lingual, and (Y) labial views. (Z–DD) ALMNH 5360, paratype, upper right posteriorly situated lateroposterior tooth in (Z) lingual, (AA) labial, (BB) mesial, (CC) oral, and (DD) basal views. Scale bars equal 1.0 cm.

*Cretalamna bryanti* Ebersole & Ehret sp. nov.
Figs. 1, 5–7
**LSID:** urn:lsid:zoobank.org:act:1D850914-5CE2-4243-A0D9-0088B7046B13
2007 *Serratolamna serrata* (Agassiz, 1843); Shimada & Brereton, 2007: 106–110, fig. 2a–g.

**Etymology**

*bryanti* = in honor of the Bryant family, whose commitment to education and ongoing support of the University of Alabama, the ALMNH, and MSC have enhanced the reputations and missions of all three institutions.

**Holotype**

MSC 2984.1, upper right lateroposterior tooth (Figs. 1, 7A–7E).

**Type locality**

Site ADa-3, Dallas County, Alabama, USA (Figs. 2 and 4). See Appendix 1 for additional locality information.

**Type horizon**

Lower Mooreville Chalk, early, but not earliest, Campanian, *Globotruncanita elevata* Brotzen, 1934 Primary Foraminifera Range Zone (see Fig. 3), upper half of the *Acuminobrachycythere acuminata* Hazel & Paulson, 1964 Ostracode Zone, Nannofossil Zone CC-18a (see Appendix 1).

**Paratypes**

ALMNH 3322, upper right anterior tooth (Figs. 5A–5E); ALMNH 6306, lower left anteriorly situated lateroposterior tooth (Figs. 6Z–6DD).

**Additional Material**

30 teeth: ALMNH 1068, ALMNH 1164, ALMNH 1407, ALMNH 1682, ALMNH 1719, ALMNH 3330 (Figs. 6A–6E), ALMNH 3331, ALMNH 3566 (Figs. 5F–5J), ALMNH 3935 (Figs. 5K–5O), ALMNH 4190 (Figs. 7U–7Y), ALMNH 4517 (Figs. 7F–7J), ALMNH 5195.1, ALMNH 5195.2, ALMNH 5360 (Figs. 7Z–7DD), ALMNH 6728, ALMNH 6760 (Figs. 7K–7O), ALMNH 8668, ALMNH 9216 (Figs. 6K–6O), ALMNH 9348, ALMNH 9724 (Figs. 5P–5T), ALMNH 9878, ALMNH 15245, ALMNH 15250, MSC 1139.9, MSC 5698, MSC 5768, MSC 26121 (Figs. 6U–6Y), MSC 34051 (Figs. 6F–6J), MSC 37499 (Figs. 7P–7T), MSC 37711 (Figs. 6P–6T). See Table 1 for detailed specimen information.

**Diagnosis**

Anterior teeth with a tall triangular main cusp, and single pair of divergent, lanceolate, lateral cusplets. Small secondary cusplets may be present on larger anterior teeth. Pronounced triangular root boss present with deep U-shaped basal concavity. Root lobes are long, range from rounded to angular (but are often angular distally), and are not as divergent as other *C. appendiculata*-like taxa. Anteriorly situated lateroposterior teeth have tall, triangular, distally inclined or distally hooked main cusp, and V-shaped neck (collar). One to two pairs of divergent, lanceolate, lateral cusplets present. Roots are symmetrical in basal view. Basal concavity is U-shaped. Lateroposterior teeth have a triangular main cusp with a strong distal hook, one to two pairs of divergent and lanceolate lateral cusplets, and labially inclined roots. Root lobes are scalloped on some teeth, and all have a shallow U-shaped basal concavity.

### Description

#### Anterior teeth

Paratype specimen ALMNH 3322 (Figs. 5A–5E) best exemplifies the teeth in the anterior files. Identified as belonging to an upper right anterior position, this tooth measures 14.4 mm in greatest mesiodistal width and 23.8 mm in greatest apicobasal height (Table 1). The main cusp on the tooth is tall, triangular, and erect. In lingual and labial views, the distal edge of the main cusp is straight, while the upper two-thirds of the mesial edge is slightly convex. The basal half of the main cusp on the labial face is slightly convex and the apical half is flat. A shallow sulcus is present medially at the base of the main cusp on the labial face. The tooth has a single pair of strongly divergent cusplets. The cusplets have a slightly lanceolate outline in labial and lingual views. The labial and lingual faces of the cusplets are convex, but more so lingually. A shallow V-shaped neck (collar) is present at the lingual crown base. The mesial and distal cutting edges extend onto, and across, the lateral cusplets. The root lobes are rounded and divergent, with the mesial lobe being slightly more elongated than the distal lobe. The basal concavity is deep and U-shaped. A pronounced, triangular, lingual protuberance is present on the root. There is no nutritive groove, but several minute nutritive foramina are visible on the lingual root protuberance.

Overall, the main cusp on the anterior teeth tend to have a slightly increasing degree of distal inclination the closer they are positioned to the commissure (Fig. 5). Based on comparisons with a jaw set from the Recent *Isurus paucus* Guitart, 1966, both upper and lower anterior teeth are present within our sample. In profile view the upper anterior teeth can be distinguished by having a somewhat flat labial face on the main cusp and a labially bent apex (Figs. 5A–5O). In contrast, the labial face of the main cusp on the lower anterior teeth range from flat to having a slight lingual bend (Figs. 5P–5T). One tooth in our sample, ALMNH 3935 (Figs. 5K–5O), has a small secondary cusplet visible on the lateral edge of the medial cusplet. This specimen is the largest anterior tooth in our sample, measuring 33.8 mm in overall height (see Table 1), suggesting that the acquisition of secondary cusplets on the anterior teeth could be attributed to ontogeny.

#### Anteriorly situated lateroposterior teeth

Paratype specimen ALMNH 6306 (Figs. 6Z–6DD) best illustrates the anteriorly positioned lateroposterior teeth of *C. bryanti* sp. nov. This specimen measures 20.8 mm in total height and 18.5 mm in greatest mesiodistal width (Table 1). The main cusp on the tooth is tall and triangular. The mesial edge of the main cusp is slightly convex and the distal edge is concave, forming a distally inclined cusp. The labial and lingual faces of the main cusp are convex, but more so lingually. A pair of prominent lanceolate cusplets is present, and a minute secondary cusplet is visible on both the mesial and distal sides. This secondary cusplet appears under-developed and is largely united to the much larger medial cusplet. The primary cusplets are strongly divergent. The mesial and distal cutting edges extend to the base of the main cusp and across the lateral cusplets. The lingual crown face is smooth and a shallow V-shaped neck (collar) is present at the crown base. The labial crown face is also smooth. The root lobes are slightly divergent, labiolingually compressed, and the mesial root lobe is mesiodistally not as wide as the distal lobe, both of which are sub-angular. A deep U-shaped basal concavity is present, as is a lingual protuberance that is triangular in basal view. The root is nearly symmetrical in basal view. No nutritive groove is present but a series of minute nutritive foramina are located on the lingual root protuberance.

The anteriorly situated lateroposterior teeth appear to bear the characteristics of both the anterior and lateroposterior tooth morphologies. The presence of small secondary cusplets on many of these teeth appear to serve as a transition from the single-cuspleted anterior teeth (Fig. 5) to the often double-cuspleted lateroposterior teeth (Fig. 7). These teeth also have a distally inclined main cusp that is like those in the lateroposterior files, but differ by having a taller cusp (see Table 1) and by lacking a labially inclined root (compare profile views on Figs. 6–7). Although the labial crown face is smooth on most anteriorly situated lateroposterior teeth, faint enameloid folding is present at the base of the main cusp on some teeth (see Fig. 6X).

Several teeth in our sample lack secondary sets of lateral cusplets (see Figs. 6A–6E, 6U–6Y). On these teeth, the lateral edge on the primary set of cusplets is slightly extended laterally, likely homologous to a secondary cusplet. At times, a secondary cusplet is present mesially, but absent distally (Figs. 6F–6J). Upper teeth in our sample were differentiated by having a main cusp with a slight labial bend (Figs. 6A–6O, 6U–6Y). In contrast, the lower teeth have a cusp that ranges from flat to a having a slight lingual bend (Figs. 6Z–6DD).

Seven teeth in our sample (ALMNH 1164, ALMNH 5195.1-2, ALMNH 6728, ALMNH 8668, ALMNH 9216, and MSC 37711) are similar in overall morphology to those described above, but are larger in overall size and have a wider cusp base (Figs. 6K–6T, see Tab.1). The secondary set of lateral cusplets are often more defined on these larger teeth, and a minute denticle is at times present between the larger mesial cusplet and the base of the main cusp (Figs. 6P–6Q). Due to the large size of these teeth (up to 27 mm in height; see Table 1), we attribute these morphological differences to ontogeny and suggest that the cusp base gets wider and the secondary cusplets more defined as the shark reaches its maximum size.

#### Lateroposterior teeth

The teeth described below, referred herein as lateroposterior teeth, likely occupy the middle third of the inferred lateroposterior hollow. Teeth within this tooth group are best illustrated by the holotype, MSC 2984.1 (Figs. 1, 7A–7E), which is assigned here to an upper right lateroposterior tooth. This tooth measures 17.6 mm in greatest mesiodistal width and 16.7 mm in greatest apicobasal height (Table 1). The main cusp on the tooth is tall and triangular and has a strong distal hook due to having a very convex mesial edge and weakly concave distal edge. The lingual face of the main cusp is smooth and convex, whereas the labial face has a slight convexity and a small number of indistinct enameloid folds is present at the base. Two pairs of divergent lateral cusplets are present, with the medial pair being more than twice the height of the lateral set. The smaller, lateral pair of cusplets are largely separated from the medial pair. The lateral cusplets are lanceolate and have slightly convex mesial and distal edges. The mesial and distal cutting edges extend continuously from the apex of the main cusp across both pairs of lateral cusplets. A shallow V-shaped neck (collar) is present at the lingual crown base. The root lobes are angular and the base of the lobes are scalloped. The basal face of the root is flat, and the root has a distinct labial bend in profile view (see Fig. 7). The root has a shallow U-shaped basal concavity and a pointed lingual protuberance that is well demarcated in basal view. There is no nutritive groove, but a series of small nutritive foramina are located on the lingual protuberance.

Overall, the main cusp on the lateroposterior teeth appear to become slightly more distally hooked the closer they are positioned to the commissure. Due to the similarity of the lateroposterior teeth in our sample, they are interpreted to belong to the upper dentition as all have a straight to labially bent main cusp in profile view. The number of lateral cusplets is variable and ranges from three to four (at times a secondary cusplet is visible on the distal side only). One tooth in our sample, MSC 37499 (Figs. 7P–7T), is unique by having both a mesiodistally wider cusp base (10.9 mm vs. 7.1 to 9.3 mm) and much broader distal root lobe (the mesial lobe may also be expanded, but is not preserved). Despite having a wider cusp base, the crown height of MSC 37499 (12.8 mm) falls within the range of the other lateroposterior teeth in our sample (8.9 to 14.1 mm, see Table 1). It is our interpretation that this tooth represents a large, mature morphology and suggests that the cusp base and root lobes on the teeth get wider as the shark approaches its maximum size.

## Remarks

Shimada & Brereton (2007) referred six teeth from our sample, MSC 1615.1, MSC 2984.1 (Figs. 1, 7A–7E), MSC 5698, MSC 5706, MSC 5768, and ALMNH 1164 (listed as PV 1988.20.73.4), to the Late Cretaceous taxon *Serratolamna serrata* (Agassiz, 1843). The teeth of *Cretalamna bryanti* sp. nov., however, differ appreciably from the type specimens of *S. serrata* illustrated by Agassiz (1843, vol. 3, pl. 32: 27–28) by lacking an elongated distal root lobe (which is present on all *S. serrata* teeth, regardless of position), by having one to two pairs of mesial and distal cusplets (lateral teeth of *S. serrata* generally have three distal and two mesial cusplets), and by lacking a nutritive groove (which is prominent on all *S. serrata* teeth; see Arambourg, 1952, pl. 15, figs. 1–41; Welton & Farish, 1993, p. 112, fig. 1–6).

As part of a later study (i.e., Ikejiri et al., 2013), one of the present authors (JE) examined several teeth with the *C. bryanti* sp. nov. morphology but interpreted them as representing large lateral teeth belonging to *Cretalamna appendiculata* (Agassiz, 1843). However, Siversson et al.’s (2015) study of *C. appendiculata*-like taxa lent clarity to the morphology of *C. appendiculata* (*s.s.*) teeth, solidifying to the present authors that the Alabama teeth do not belong to this latter taxon. Not only can the teeth of *C. bryanti* sp. nov. grow to much larger sizes (up to 27 mm in overall height; see Table 1), but the teeth of *C. appendiculata* (*s.s.*) never have more than a single pair of lateral cusplets (see Siversson et al., 2015, fig. 2). In addition, the main cusps on the anterior teeth of *C. bryanti* sp. nov. are much taller, more triangular, and have less bi-convex mesial and distal edges. Finally, the main cusps on the lateroposterior teeth of *C. bryanti* sp. nov. are much more distally hooked than those on *C. appendiculata* (*s.s.*).

Siversson et al. (2015) described numerous *Cretalamna*-like taxa, all but two of which could be placed into the *Cretalamna appendiculata, Cretalamna borealis,* or *Cretalamna hattini* species groups. Of these, the teeth of *Cretalamna bryanti* sp. nov. appear best aligned with the *C. borealis* species group which includes *Cretalamna borealis* Priem, 1897*, Cretalamna ewelli* Siversson et al., 2015, and *Cretalamna gertericorum* Siversson et al., 2015. Characteristics shared with the *Cretalamna borealis* species group include: (1) the presence of slender and elongated anterior teeth; (2) moderately wide lateroposterior teeth (which are mesiodistally much wider in the *C. appendiculata* and *C. hattini* groups); (3) a pronounced and well demarcated lingual protuberance on the root (especially apparent in basal view); (4) convex labial and lingual faces on the main cusp; (5) the unusual degree of distal cusp inclination on lateroposterior teeth; (6) the divergent and lanceolate shape of the lateral cusplets; (7) the presence of two pairs of lateral cusplets on some teeth; (8) the presence of scalloped root lobes on some teeth; and (9) the roots on the lateroposterior teeth often have a symmetrical sub-rectangular outline (Fig. 6).

In a comparison with the other members of the *C. borealis* species group, the anterior teeth of *C. bryanti* sp. nov. differ in several respects. First, in basal view, the lingual root protuberance on the anterior teeth of *C. bryanti* sp. nov. is generally more triangular and pronounced (Fig. 5) than on *C. borealis* (Siversson et al., 2015, figs. 9–10), *C. ewelli* (Siversson et al., 2015, fig. 12) or *C. gertericorum* (Siversson et al., 2015, fig. 11). Second, in labial or lingual views, the root lobes are less divergent (Fig. 5) than those on *C. borealis*, *C. ewelli,* or *C. gertericorum*, and the root lobes are longer than those on *C. borealis* and *C. ewelli*. Third, the basal concavity is more U-shaped on the anterior teeth of *C. bryanti* sp. nov., while slightly more V-shaped on *C. borealis*, *C. ewelli,* or *C. gertericorum* (a product of having more divergent root lobes). The basal concavity is also deeper on the anterior teeth of *C. bryanti* sp. nov. than on those on *C. borealis* and *C. ewelli*. Other differential characteristics on the *C. bryanti* sp. nov. anterior teeth include having a wider and more triangular main cusp than on *C. ewelli* and *C. gertericorum*, and a distal root lobe that is more angular than on *C. borealis.* Furthermore, the anterior teeth of *C. bryanti* sp. nov. have wider lateral cusplets and a more V-shaped neck (collar) than on *C. ewelli*. Moreover, the anterior teeth of *C. bryanti* sp. nov. have a broader and more triangular cusp when compared to similar teeth of the same size. In addition, when compared to the other *C. borealis* group species, the height of the root is greater relative to the height of the crown*.* Finally, the upper anterior teeth of *C. bryanti* sp. nov. have the unique combination of a relatively straight cusp that is labially inclined. This combination of characteristics has only been observed on the probable upper 3rd anterior position of *C. borealis* and *C. ewelli* (Siversson et al., 2015, fig. 12E).

The lateroposterior teeth of *C. bryanti* sp. nov. (Fig. 7) can be separated from those of *C. ewelli* by having three to four lateral cusplets on nearly all the specimens examined (as opposed to two on *C. ewelli*), a wider main cusp that is more distally hooked and has a more convex mesial edge, root lobes that are generally more angular, and a lingual neck (collar) that is more V-shaped. The *C. bryanti* sp. nov. lateroposterior teeth differ from those of *C. gertericorum* by having a slightly taller crown, a more triangular chevron, and the presence of two pairs of lateral cusplets on nearly all the teeth examined. A secondary set of lateral cusplets is present on only one of the *C. gertericorum* teeth figured by Siversson et al. (2015, fig. 11E3), a possible commissural tooth, but it is larger and less defined than those observed on *C. bryanti* sp. nov. (although this latter observation may be due to wear). When compared to those of *C. borealis*, the lateroposterior teeth of *C. bryanti* sp. nov. differ by having a more pronounced and triangular root boss, a less rectangular root outline in basal view, a basal concavity that is more U-shaped, a more V-shaped neck (collar), and a taller, more distally hooked main cusp.

Unfortunately, Siversson et al. (2015) did not figure any anteriorly situated lateroposterior teeth for *C. borealis*, *C. ewelli,* or *C. gertericorum,* negating a direct comparison for all three of these taxa with those in our sample. However, a photograph obtained of a *C. borealis* tooth (WAM 13.5.22) from Sweden compares favorably with one tooth in our sample, MSC 26121 (Figs. 6U–6Y), as both are likely from the upper first anteriorly situated lateroposterior position. Specimen MSC 26121 differs from this *C. borealis* tooth by having a more distally inclined main cusp, more divergent lateral cusplets, and a more U-shaped basal concavity.

At least two additional undocumented species of *Cretalamna* (*s.s.*) have been observed by the lead author (JE) to be present within Late Cretaceous deposits in Alabama. Both of these taxa have a cusp that is much shorter than those on *C. bryanti* sp. nov., they have a higher root relative to the height of the crown, a main cusp that is less distally inclined in the lateroposterior positions, and the teeth never have more than a single pair of lateral cusplets. These two taxa likely belong to Siversson et al.’s (2015) *C. appendiculata* or *C. hattini* species groups.

Elsewhere in the literature, Lauginiger & Hartstein (1983, p. 31, pl. 2, figs. 11–14, text fig. 13) described and figured teeth from the middle Maastrichtian Mt. Laurel Formation of Delaware that have a similar morphology to those of *C. bryanti* sp. nov. Originally assigned by Lauginiger & Hartstein (1983, pl. 2, figs. 12–13) to the subspecies “*Cretolamna appendiculata pachyrhiza*” (Herman, 1977), these teeth were later synonymized with *C. borealis* by Siversson et al. (2015). The teeth figured by Lauginiger & Hartstein (1983) differ from those of *C. bryanti* sp. nov. by having root lobes that are more rounded and more divergent, creating a wider basal concavity (Lauginiger & Hartstein, 1983, pl. 2, figs. 11–14). Furthermore, the lateral cusplets on the anteriorly situated lateroposterior and lateroposterior teeth of *C. bryanti* sp. nov. are much more separated from the main cusp than those figured by Lauginiger & Hartstein (1983), and none of the figured lateroposterior teeth have a secondary pair of lateral cusplets.

Arambourg (1952, fig. 14) figured 24 teeth from the Maastrichtian (Cretaceous) and Danian (Paleocene) of Morocco, several of which (figs. 14.1–11) appear superficially similar to the lateroposterior teeth of *C. bryanti* sp. nov. by having a laterally hooked main cusp and two pairs of lateral cusplets. However, the lateroposterior teeth of *C. bryanti* sp. nov. differ by generally having a slightly more distally hooked main cusp, a more concave labial crown face, and roots lobes that are more labially inclined. In addition, the dentition of *C. bryanti* sp. nov. differs by having anteriorly situated lateroposterior teeth with a hooked or distally inclined main cusp, while those illustrated for *C. arambourgi* are erect (see Arambourg, 1952, figs. 16–18). The anterior teeth associated with *C. arambourgi* also differ by having a much more pronounced and demarcated lingual protuberance (see Arambourg, 1952, figs. 12–13), and a main cusp that is shorter, mesiodistally narrower, and more bi-convex than those of *C. bryanti* sp. nov. Subsequently, these teeth, which were originally referred to “*Lamna appendiculata*” by Arambourg (1952), were assigned to a new subspecies, “*Cretolamna appendiculata arambourgi”,* by Cappetta & Case (1975), and later elevated to species level (i.e., *C. arambourgi*) by Siversson et al. (2015). The similarity of this taxon to *C. bryanti* sp. nov., as well to the other members of the *C. borealis* group, suggests that *C. arambourgi* may also have affinities with this species group.

### Stratigraphic and geographic distribution of *Cretalamna bryanti* sp. nov.

The specimens in our sample were all collected from two Upper Cretaceous stratigraphic units in Alabama, the Tombigbee Sand Member of the Eutaw Formation and the overlying Mooreville Chalk (Fig. 3). In Alabama, the contact between the Tombigbee Sand Member and the Mooreville Chalk is time transgressive and straddles the Santonian/Campanian boundary. In central Alabama this contact falls within the upper Santonian, but in the western part of the state, the contact resides within the lower Campanian (Mancini, Puckett & Tew, 1996; Puckett, 2005). One specimen in our sample, MSC 37499 (Figs. 7P–7T), was derived from the lower unnamed member of the Mooreville Chalk at site AMg-1 in Montgomery County (Fig. 2). Collected from central Alabama, this tooth represents the stratigraphically oldest *C. bryanti* sp. nov. specimen within our sample, with an age that falls within the upper Santonian *Dicarinella asymetrica* Planktonic Foraminiferal Zone (see Fig. 3; Puckett, 2005), the last occurrence of which defines the Santonian/Campanian boundary (Caron, 1985). The remainder of the specimens were derived from Tombigbee Sand Member and Mooreville Chalk exposures in Dallas, Greene, and Hale counties, all located in west-central or western Alabama. In these parts of the state, the exposures of the Tombigbee Sand Member and Mooreville Chalk both fall within the early Campanian *Globotruncanita elevata* Planktonic Foraminiferal Zone (see Fig. 3), providing a bracketed late Santonian to early Campanian age for *C. bryanti* sp. nov. in Alabama, with all the specimens being derived from the *D. asymetrica* and *G. elevata* Planktonic Foraminiferal Zones.

In east Alabama, the Tombigbee Sand Member and Mooreville Chalk grade into the Santonian/Campanian Blufftown Formation. Although the Blufftown Formation is stratigraphically equivalent to the Tombigbee Sand Member and Mooreville Chalk of central and west Alabama, no teeth with the *C. bryanti* sp. nov. morphology were reported within the only published chondrichthyan study conducted within this formation (see Case & Schwimmer, 1988). The present absence of *C. bryanti* sp. nov. from the Blufftown Formation is likely a result of historical under-sampling within this unit, leading to a paucity of specimens being reposited in scientific collections.

*C. bryanti* sp. nov. specimens are also conspicuously absent from the Santonian, non-Tombigbee Sand Member component of the Eutaw Formation in Alabama, as well as from the upper Campanian Demopolis Chalk and the Campanian/Maastrichtian Ripley Formation, and Maastrichtian Prairie Bluff Chalk, and Providence Sand. However, in Alabama, field collecting within the non-Tombigbee component of the Eutaw Formation and the Demopolis Chalk has yielded far fewer vertebrate remains than the Tombigbee Sand Member or Mooreville Chalk (see Russell, 1988; Ikejiri et al., 2013). Furthermore, all the Maastrichtian deposits in the state have traditionally been under-sampled. Thus, it is our conclusion that collecting and preservation biases likely play a role in the limiting the stratigraphic range of *C. bryanti* sp. nov. in the state.

The present authors are not aware of any teeth with the *C. bryanti* sp. nov. morphology to have been figured or described elsewhere in the literature. However, it is possible that the previous lack of recognition of this morphology is the result of the misidentification of isolated teeth and their subsequent referral to other similar taxa. Thus, it is our conclusion that the geographic range of this taxon is likely much more extensive than reported here and deposits bearing similar age chondrichthyan assemblages may yield additional specimens.

## Discussion

The recognition of *C. bryanti* sp. nov. from Alabama adds to our knowledge on the diversity of the Late Cretaceous members of *Cretalamna* (*s.s*.) and the Otodontidae. Understanding the relationship of the Otodontidae and the Cretoxyrhinidae within the Lamniformes has been a problematic subject for decades due to misidentification of type specimens (for example, see above for a discussion on Agassiz’s *Cretalamna appendiculata* type series) and disagreements about the taxonomy of various genera including *Carcharodon*, *Otodus*, *Cretalamna*, and *Cretoxyrhina*. The latter disagreements stem mainly from the poor preservation of shark cartilage, leading to a fossil record for the Chondrichthyes that is composed largely of isolated teeth.

Historically, certain members of the Otodontidae (i.e., *Otodus* (*Carcharocles*) and *Otodus* (*Megaselachus*)) were placed within the genus *Carcharodon* within the Lamnidae, and *Cretalamna* was placed within the Cretoxyrhinidae along with *Cretoxyrhina* and other assorted taxa (Agassiz, 1843; Cappetta, 1987; Applegate & Espinosa-Arrubarrena, 1996). In 1964, Glickman proposed that the mega-toothed sharks should be removed from the Lamnidae and placed within their own family. Subsequently, the limited distribution of his book (which was published in Russian) and the radicalness of his idea initially led to the lack of widespread acceptance of his proposal. However, new discoveries within the past 20 years has led to the majority acceptance of Glikman’s (1964) assertion that the mega-toothed sharks (i.e., the *Otodus* lineage) were a separate lineage from the lamnids and should be placed within the Otodontidae (Zhelezko & Kozlov, 1999; Cappetta, 2012; Ehret et al., 2012; Siversson et al., 2015). The Cretoxyrhinidae has since been shown to be a polyphyletic ‘wastebasket’ taxon (Siversson et al., 2015), and *Cretalamna* (*s.s*.) is currently recognized as belonging within the Otodontidae, having closer affinities to *Otodus* than to *Cretoxyrhina* (Siverson, 1999; Underwood & Cumbaa, 2010; Siversson et al., 2015).

The appearance of *Otodus obliquus* (Agassiz, 1843) in the Danian (Early Paleocene) of Alabama (see Ehret & Ebersole, 2014), and its morphological similarity with some of the Late Cretaceous *Cretalamna* (*s.s*.) taxa, certainly suggests that one of the species groups reported by Siversson et al. (2015) gave rise to the *Otodus* lineage. However, due to the complexity of the *Cretalamna* (*s.s*.) species complex and the likelihood that numerous Late Cretaceous members are yet to be described, at present it is not possible to ascertain for certain from which lineage *Otodus* may have been derived. Hopefully future studies on Late Cretaceous *Cretalamna* (*s.s*.) diversity will shed light on these complex evolutionary relationships.

## Conclusions

Siversson et al.’s (2015) study of Late Cretaceous *Cretalamna*-like taxa has led to the present recognition of a new species of *Cretalamna* (*s.s.*) from Alabama, *Cretalamna bryanti* sp. nov. Multiple characteristics align this new taxon with Siversson et al.’s (2015) *Cretalamna borealis* species group including: elongated anterior teeth; a pronounced and demarcated lingual root protuberance; convex labial cusp face; strong distal cusp inclination in upper lateroposterior files; up to two pairs of divergent, lanceolate lateral cusplets; scalloped roots lobes on some teeth; and roots on lateroposterior teeth that are largely symmetrical in basal view. The anterior teeth of *C. bryanti* sp. nov. differ from the other members of this group by having root lobes that are less divergent (forming a more U-shaped basal concavity); and an erect main cusp on upper anterior teeth that are also labially inclined. The anteriorly situated lateroposterior and lateroposterior teeth differ by the presence of three to four well-defined lateral cusplets on most teeth in combination with a laterally inclined or strong distally hooked main cusp.

Our sample of *C. bryanti* sp. nov. teeth indicate that the dentition of this shark had varying degrees of monognathic, disjunct, and dignathic heterodonty, and the presence of a few larger teeth with slight morphological differences suggests a degree of ontogenetic heterodonty was present as well. At this time, *C. bryanti* sp. nov. appears stratigraphically confined to the Santonian/Campanian *Dicarinella asymetrica* and *Globotruncanita elevata* Planktonic Foraminiferal Zones within the Tombigbee Sand Member of the Eutaw Formation and Mooreville Chalk in Alabama. The depositional settings for these units suggest *C. bryanti* sp. nov. favored low energy shallow, middle shelf, environments. In Alabama, this species is currently confined to four counties in the central and western parts of the state, but the absence of this taxon from other counties and stratigraphic units could be the result of collecting and preservation biases. The lack of recognition of this morphology elsewhere is likely the result of the misidentification of isolated teeth and their subsequent referral to morphologically similar taxa.

The recognition of *C*. *bryanti* sp. nov. from Alabama adds to our knowledge on the diversity and distribution of otodontids in North America. With the presence of several recently recognized species of Late Cretaceous *Cretalamna* (*s.s*.) in the United States (see Siversson et al., 2015), and recently described Paleogene otodontids from Alabama (Ehret & Ebersole, 2014), we are beginning to gain clarity on the diversity and distribution of otodontids in the region.

## Institutional abbreviations

ALMNH: Alabama Museum of Natural History, University of Alabama, Tuscaloosa, USA
MSC: McWane Science Center, Birmingham, Alabama, USA
WAM: Western Australia Museum, Welshpool

## Appendix 1. *Cretalamna bryanti* sp. nov. collecting localities

*Locality*: Site ADa-3 (Type Locality), “Harrell Station”, Dallas County, Alabama, coordinates: 32.422029, −87.201061.

*Exposed stratum*: Lower Mooreville Chalk.

*Stratigraphic Age*: Lower, but not lowest Campanian, *Globotruncanita elevata* Brotzen, 1934 Primary Foraminifera Range Zone, upper half of the *Acuminobrachycythere acuminata* Hazel & Paulson, 1964 Ostracode Zone, Nannofossil Zone CC-18a.

*Remarks*: Known locally as Harrell Station, this 140-acre plot of land is property of the Alabama Museum of Natural History at the University of Alabama, Tuscaloosa. This locality consists of extensive gully exposures of Mooreville Chalk. No other marine strata are exposed at this locality. Prior analyses of the ostracodes, planktonic foraminifera, and nannoflora by Puckett (2005) and Prieto-Márquez, Erickson & Ebersole (2016b) have shown that Mooreville Chalk exposures at site ADa-3 fall within the early, but not earliest, Campanian. Nearly half (15 of 33) of the specimens in our sample, the type material included, were collected from this locality. All were obtained via surface collection. Site ADa-3 is also the type locality for the hadrosauroid *Lophorhothon atopis* Langston, 1960, and numerous fishes and marine turtles (see Applegate, 1970; Zangerl, 1953a; Zangerl, 1953b; Zangerl, 1960).

*References*: Zangerl (1948), Zangerl (1953a), Zangerl (1953b), Zangerl (1960), Applegate (1970), Puckett (2005), Prieto-Márquez, Erickson & Ebersole (2016b).

*Locality*: Site ADa-19, Dallas County, Alabama. More detailed locality information is on file at the ALMNH and MSC.

*Exposed strata*: Upper Tombigbee Sand Member of the Eutaw Formation, lower Mooreville Chalk.

*Stratigraphic Age*: Lower Campanian, Lower *Globotruncanita elevata* P.R.Z.

*Remarks*: Located on private land, this locality is situated on the right bank of the Cahaba River, just south of an unnamed iron bridge. Two specimens in our sample were historically collected from this unpublished locality; however, a stratigraphic section has yet to be produced for the site. The present authors have not visited this locality, but according to unpublished historical field notes on file at MSC (entry 57-02), exposures of both the upper portion of the Tombigbee Sand Member of the Eutaw Formation and lower Mooreville Chalk are present. Data associated with the two collected specimens, ALMNH 4517 and ALMNH 3331, indicate they were derived from the Mooreville Chalk exposures located at the top of the exposed section. The method of collection was not recorded with the specimens.

*References*: Unpublished locality.

*Locality*: Site ADa-20, Dallas County, Alabama. More detailed locality information is on file at the ALMNH and MSC.

*Exposed stratum*: Lower Mooreville Chalk.

*Stratigraphic Age*: Lower Campanian, Lower *Globotruncanita elevata* P.R.Z.

*Remarks*: Two specimens, ALMNH 4517 and ALMNH 3331, were historically collected from this unpublished locality. The present authors have not visited this locality and a stratigraphic section has yet to be produced for the site. Unpublished site log data on file at both the ALMNH and MSC (entry ADa-20) indicates that this locality consists of a small batch of erosional Mooreville Chalk gullies that is located on private land. Data associated with the specimens confirms that they were derived from the Mooreville Chalk, and with this locality located less than half a mile north of site ADa-3, the exposures are of approximate age. The method of collection was not recorded with the specimens, but they were likely surface collected. No other Cretaceous strata are recorded to be exposed at this locality.

*References*: Unpublished locality.

*Locality*: Site AGr-4, Greene County, Alabama. More detailed locality information is on file at the ALMNH and MSC.

*Exposed stratum*: Lower Mooreville Chalk.

*Stratigraphic Age*: Lower Campanian, Lower *Globotruncanita elevata* P.R.Z.

*Remarks*: Site AGr-4, which is located on private land, is the type locality for the Late Cretaceous enantiornithine bird, *Halimornis thompsoni* Chiappe, Lamb Jr & Ericson (2002) as well as the collecting site for a referred plesiosaur specimen *Polycotylus latipinnis* described by O’Keefe (2004). This locality consists of a series erosional Mooreville Chalk gullies that Chiappe, Lamb Jr & Ericson (2002) interpreted to be late-early to early-middle Campanian in age. Two specimens were surface collected from this locality, ALMNH 6306 and MSC 26121.

*References*: Chiappe, Lamb Jr & Ericson (2002), O’Keefe (2004).

*Locality*: Site AGr-5, Greene County, Alabama. More detailed locality information is on file at the ALMNH and MSC.

*Exposed stratum*: Lower unnamed member of the Mooreville Chalk.

*Stratigraphic Age*: Lower Campanian, Lower *Globotruncanita elevata* P.R.Z.

*Remarks*: This unpublished locality is located on private land and consists of a large gully exposure of Mooreville Chalk. Three specimens were historically collected from this locality, ALMNH 3935, ALMNH 5195.1, and ALMNH 5195.2, however no stratigraphic profile of the site has been produced. Although the present authors have not visited this locality, detailed unpublished field notes on file at MSC (entry 114-01), and unpublished site log data on file at both the ALMNH and MSC (entry AGr-5), both indicate the lower unnamed member of the Mooreville Chalk is exposed at this locality. The specimens obtained from this locality were likely surface collected.

*References*: Unpublished locality.

*Locality*: Site AGr-18, Greene County, Alabama. More detailed locality information is on file at the ALMNH and MSC.

*Exposed stratum*: Lower Mooreville Chalk.

*Stratigraphic Age*: Lower Campanian, Lower *Globotruncanita elevata* P.R.Z.

*Remarks*: One specimen, MSC 5698, was historically surface collected from this unpublished locality. This site consists of a small series of erosional Mooreville Chalk gullies located on private land. The present authors have not visited this locality and no stratigraphic section of the site has been produced. However, unpublished field notes on file at MSC (entry 107-01) report that at least one of the gullies exposes the lower unnamed member of the Mooreville Chalk, indicating that all the surrounding gullies also expose the lower portion of this unit. No other Cretaceous strata are recorded to be exposed at this locality.

*References*: Unpublished locality.

*Locality*: Site AGr-43, Greene County, Alabama. More detailed locality information is on file at the ALMNH and MSC.

*Exposed stratum*: Upper Tombigbee Sand Member of the Eutaw Formation.

*Stratigraphic Age*: Lower Campanian, Lower *Globotruncanita elevata* P.R.Z.

*Remarks*: Two specimens (ALMNH 4190 and ALMNH 15250) were collected from Site AGr-43, a creek locality located on private land. Both specimens were obtained by screen washing matrix derived from gravel bars that expose the upper portion of the Tombigbee Sand Member of the Eutaw Formation. One of the present authors (JE) has observed small exposures of the overlying Mooreville Chalk at this locality, however since these exposures were located more than 0.8 km downstream from the main collecting areas, all the specimens described within are interpreted to be derived from the Tombigbee Sand. The geologic exposures at this locality were described in detail by Becker et al. (2008) and Ciampaglio et al. (2013).

*References*: Becker et al. (2008) and Ciampaglio et al. (2013).

*Locality*: Site AHl-1, Hale County, Alabama. More detailed locality information is on file at the ALMNH and MSC.

*Exposed stratum*: Upper Tombigbee Sand Member of the Eutaw Formation.

*Stratigraphic Age*: Lower Campanian, Lower *Globotruncanita elevata* P.R.Z.

*Remarks*: A single specimen, MSC 1139.9, was historically collected from this locality. The exposed stratum within the banks of this creek site were first interpreted by Meyer (1974) to belong to the lowermost part of the Mooreville Chalk. However, later visits to the site by the current authors have confirmed that the exposures belong to the upper 4.5 m of the underlying Tombigbee Sand Member of the Eutaw Formation (ALMNH and MSC site locality entry AHl-1). The present authors have also confirmed that the upper Tombigbee Sand is the only Late Cretaceous unit exposed at this locality. The lone specimen reported here is was historically collected, but likely picked from screen washed matrix derived from gravel bars at the site. This site is located on private land.

*References*: Meyer (1974).

*Locality*: Site AMg-1, Montgomery County, Alabama. More detailed locality information is on file at the ALMNH and MSC.

*Exposed strata*: Upper Tombigbee Sand Member of the Eutaw Formation, lower unnamed member of the Mooreville Chalk.

*Stratigraphic Age*: Upper Santonian, upper *Dinarinella asymetrica* T.R.Z.

*Remarks*: A single specimen, MSC 37499, was historically collected from this locality. Site AMg-1 is the type locality for the hadrosauroid, *Eotrachodon orientalis* (Prieto-Márquez, Erickson & Ebersole, 2016a). This creek locality exposes both the upper portion of the Tombigbee Sand Member of the Eutaw Formation and the overlying lower unnamed member of the Mooreville Chalk. Data associated with specimen MSC 37499 indicates it was derived from the exposures of the lower unnamed member of the Mooreville Chalk. The geology of this locality was described in detail by Prieto-Márquez, Erickson & Ebersole (2016b) and an unpublished stratigraphic profile is on file within the locality files at both the ALMNH and MSC (entry AMg-1).

*References*: Prieto-Márquez, Erickson & Ebersole (2016b).

*Localities:* AGr-Exp. 33, AGr-Exp. 35.

*Exposed strata*: Mooreville Chalk.

*Stratigraphic Age*: Lower Campanian, Lower *Globotruncanita elevata* P.R.Z.

*Remarks*: The precise collecting localities for three specimens in our sample is unclear. Two specimens, ALMNH 6728 and ALMNH 6760, were collected during the 2003 ALMNH Summer Expedition, while ALMNH 9348 was collected during the 2005 ALMNH Summer Expedition. These annual summer expeditions are open to the general public, but are led by ALMNH personnel. During the summers of 2003 and 2005, collecting trips were led to various localities in Greene County, including site AGr-43 (which exposes the Tombigbee Sand Member of the Eutaw Formation) and numerous gully sites near Clinton, Alabama (which all expose sections of Mooreville Chalk). Chalk is still present on the roots of all three specimens, indicating they were surface collected from the Mooreville Chalk exposures in the area. The ALMNH and MSC locality files record numerous gully sites in the area, making it unclear which particular locality the specimens were derived. As a result, the collecting localities for these specimens have been designated as AGr-Exp. 33 or AGr-Exp. 35. However, all the of the gully localities in this area are lithologically similar to site AGr-18 and are equivalent in age.

*References*: Unpublished localities.

## Additional Information and Declarations

### Data Availability

The following information was supplied regarding data availability: All specimens in this study are housed in the permanent collections at the Alabama Museum of Natural History in Tuscaloosa, Alabama and McWane Science Center in Birmingham, Alabama.

### New Species Registration

The following information was supplied regarding the registration of a newly described species: Publication LSID: urn:lsid:zoobank.org:pub:F6C65E1D-19E6-4BA9-BEDD-DAFDB0BC27C9; Species name: *Cretalamna bryanti* urn:lsid:zoobank.org:act:1D850914-5CE2-4243-A0D9-0088B7046B13.
